# MmuPV1-Induced Cutaneous Squamous Cell Carcinoma Arises Preferentially from Lgr5+ Epithelial Progenitor Cells

**DOI:** 10.3390/v14081751

**Published:** 2022-08-11

**Authors:** Ruben Moreno, Darya Buehler, Paul F. Lambert

**Affiliations:** 1 McArdle Laboratory for Cancer Research, School of Medicine and Public Health, University of Wisconsin, Madison, WI 53705, USA; 2 Department of Pathology and Laboratory Medicine, School of Medicine and Public Health, University of Wisconsin, Madison, WI 53705, USA

**Keywords:** Lgr5, mouse papillomavirus, cell of origin

## Abstract

Murine papillomavirus, MmuPV1, causes natural infections in laboratory mice that can progress to squamous cell carcinoma (SCC) making it a useful preclinical model to study the role of papillomaviruses in cancer. Papillomavirus can infect cells within hair follicles, which contain multiple epithelial progenitor cell populations, including Lgr5+ progenitors, and transgenic mice expressing human papillomavirus oncogenes develop tumors derived from Lgr5 progenitors. We therefore tested the hypothesis that Lgr5+ progenitors contribute to neoplastic lesions arising in skins infected with MmuPV1 by performing lineage tracing experiments. Ears of 6–8-week-old Lgr5-eGFP-IRES-CreERT2/Rosa26LSLtdTomato mice were treated topically with 4-OH Tamoxifen to label Lgr5+ progenitor cells and their progeny with tdTomato and, 72 h later, infected with MmuPV1. Four months post-infection, tissue at the infection site was harvested for histopathological analysis and immunofluorescence to determine the percentage of tdTomato+ cells within the epithelial lesions caused by MmuPV1. Squamous cell dysplasia showed a low percentage of tdTomato+ cells (7%), indicating that it arises primarily from non-Lgr5 progenitor cells. In contrast, cutaneous SCC (cSCC) was substantially more positive for tdTomato+ cells (42%), indicating that cSCCs preferentially arise from Lgr5+ progenitors. Biomarker analyses of dysplasia vs. cSCC revealed further differences consistent with cSCC arising from LGR5+ progenitor cells.

## 1. Introduction

The first discovered papillomavirus, cottontail rabbit papillomavirus (CRPV, originally called Shope papillomavirus, recently renamed as a kappapapillomavirus), was discovered by Dr. Richard Shope in 1933 in rabbits, where it caused keratinous carcinomas [1]. CRPV became the first animal virus for studying the papillomavirus’ natural life cycle. Decades later, Dr. Stefania Jabłońska proposed the etiological association of human papillomavirus (HPV) with skin cancer, and, a few years later, Dr. Harald zur Hausen postulated that HPV, rather than the herpes virus, was responsible for cervical cancer [2,3,4,5]. This eventually led to his laboratory’s discovery of HPV16 and 18, the two strains most associated with cervical cancer. Today, approximately 5% of all human cancers are recognized to be etiologically attributable to HPV.

Despite significant advances in our understanding of the role of HPV in cancer, the study of the natural viral life cycle of papillomaviruses in vivo has been limited to laboratory animal species, for which there are identified papillomaviruses. This is because papillomaviruses are species-specific [6]. Therefore, one cannot study HPVs in vivo. Historically, studies on papillomaviruses have been performed in rabbits and cows, while for most species, papillomaviruses only cause disease within the stratified squamous epithelium, bovine papillomaviruses are classified as fibropapillomaviruses because they also cause the hyperplasia of the underlying dermal fibroblasts.

Most papillomaviruses cause disease only within stratified squamous epithelium. In cutaneous tissue, the stratified squamous epithelium is subclassified into different compartments, such as the interfollicular epidermis, sebaceous glands, and hair follicles [7]. Papillomavirus research has focused on the bulge region of the hair follicle because it contains epithelial progenitor cells that can form colonies in tissue culture with high efficiency compared to cells from other areas of the hair follicle and have the capacity for high rates of proliferation [8,9,10,11]. Specifically, in vivo, there is a burst of cell proliferation within the bulge region during the early anagen phase of the hair follicle cycle wherein bulge stem cells divide to generate transiently amplifying cells that contribute to the generation of the hair shaft [9]. In cottontail rabbits, the bulge region was shown to harbor CRPV transcripts, raising the possibility that papillomaviruses infect epithelial progenitor cells within this region of the hair follicle [12]. This discovery formed the basis for future studies on the effects that papillomaviruses have on hair follicle cells. A significant discovery utilizing lineage tracing demonstrated that, in HPV16 transgenic mice, neoplastic lesions consisted of Lgr5+ progeny [13]. Since Lgr5 serves as a marker for bulge-residing epithelial progenitor cells; this finding suggested that Lgr5+ cells likely play a role in HPV-associated pathogenesis [14,15]. Another study found an increase in K15-positive cells, a marker for Lgr5+ progeny, in the skin of transgenic mice expressing the HPV16 E7 oncogene [16]. In addition, papillomavirus can upregulate several other markers associated with increased stemness [16,17,18,19,20,21].

We utilized Lgr5-eGFP-IRES-CreER^T2^/Rosa26LSLtdTomato (hereafter referred to as Lgr5 reporter) mice to perform lineage tracing in the context of natural cutaneous infection by the recently discovered mouse papillomavirus, MmuPV1 [22]. We used this virus to discern the contributions made by Lgr5+ cells in the development of squamous dysplasia/papillomas and associated squamous cell carcinomas (SCCs) in mouse skin.

## 2. Materials and Methods

### 2.1. Animals

Lgr5-eGFP-IRES-CreER^T2^ mice were acquired from Jax labs (strain number: 008875) and generated by Dr. Hans Clevers’ lab (Hubrecht Institute, Utrecht, The Netherlands) [23]. Rosa26LSLtdTomato mice were acquired from Jax labs (strain number: 007905) [24]. Ros26LSLtdTomato mice were backcrossed onto the FVB (Taconic) background for 10 generations. Lgr5-eGFP-IRES-CreER^T2^ mice were crossed onto the FVB background for 7 generations. Sires heterozygous for Lgr5-eGFP-IRES-CreER^T2^ were bred with dames homozygous for Rosa26LSLtdTomato to generate experimental mice.

### 2.2. Virus and Infection

Crude viral extracts of MmuPV1 were isolated from papillomas on nude mice and quantified to determine the viral genome equivalents (VGE) per µL, as previously described [25]. Master stocks at 3 × 10^9^ VGE/µL were stored frozen at −80 °C and were less than a year old at the time of experimental use. Stocks were diluted in PBS to achieve a working concentration of 1 × 10^9^ VGE/µL. All mice were infected on the same day with freshly thawed aliquots from the same master stock. Anesthetized mice were lightly scarified on their ears with a 27-gauge needle, and 2 µL of virus stock was applied to the scarified area. Mock-infected mice received 2 µL of PBS instead and were housed separately from infected animals.

### 2.3. 4-OH Tamoxifen

Acetone was placed in 37 °C water bath and used to dissolve 4-OH tamoxifen at a concentration of 15 mg/mL (Sigma-Aldrich, Saint Louis, MO, USA, Cat. No. T176-50MG). This aliquot was protected from light using foil. Then, 20 µL was applied topically to each mouse ear and anesthetized mice were held for an additional 20–30 s until no visible remnants of acetone could be seen. Mice were then released back into their cages.

### 2.4. RNA In Situ Hybridization

Three representative slides were selected from three different animals based on pathological scoring in addition to two slides from mock-infected animals. In situ hybridization was conducted using the RNAscope 2.5 HD assay-brown kit (Advanced Cell Diagnostic, Newark, CA, USA) as previously described [26]. MusPV-E6-E7 probes were used to detect viral genetic material (Advanced Cell Diagnostic Newark, CA, USA, Cat No. 409771).

### 2.5. Immunofluorescence

Representative slides were selected based on pathological scoring. At least three slides, each from three different animals, were examined to select representative images for pS6, Sox9, and tdTomato images. Briefly, slides were deparaffinized in xylene, rehydrated in ethanol. Next, 3% H_2_O_2_ in methanol was applied to slides for 10 min. The slides were then boiled in 10 mM citrate buffer for 20 min (pH 6.0). Slides were then blocked with blocking buffer (Cat. No. FP1012, PerkinElmer, Boston, MA, USA) at room temperature (RT) in a humidified chamber for one hour. Slides were then incubated overnight in primary antibody at 4 °C in a humidified chamber, overnight. The next day, goat anti-rabbit-HRP secondary antibody was applied to slides (1:500). Biotin-tyramide (10 μg/mL) was then applied. AlexaFluor 488 goat anti-rabbit (Invitrogen) or Streptavidin-Alexa Fluor-594 (ThermoFisher Scientific, Waltham, MA, USA, Cat. No. S-32356) were applied as secondary antibody (at 1:1000). DAPI was also applied to slides. Slides were mounted in a prolonged diamond antifade mountant (Invitrogen Cat. No. P36970). Anti-pS6 (Cell Signaling Cat. No. 4858) was used at 1:100 dilutions. Anti-Sox9 (Abcam, Cambridge, UK, Cat No. Ab185966) was diluted at 1:400, and Tris-EDTA buffer at pH 6 was used for antigen retrieval instead of citrate buffer. Sox9 and pS6 were detected using tyramide signal amplification (TSA) [27]. Anti-K14 (Biolegend, San Diego, CA, USA, Cat No. 905301) was used at 1:1000. Anti-dsRed (Takara Cat. No. 632496) was used at 1:200 and used in conjunction with anti-K14 (ThermoFisher, Waltham, MA, USA, Cat. No. MA5-11599) at 1:500 to detect tdTomato without TSA. The same steps were followed as stated above except no treatment with H_2_O_2,_ 5% goat serum in 5% milk was used for blocking instead of PerkinElmer blocking buffer, goat anti-rabbit-HRP secondary antibody was not applied, and nor was Biotin-tyramide used.

### 2.6. tdTomatoRed Quantification

Each lesion was quantified with three images per slide across five slides within the lesion. Images were taken at 400× total magnification and K14+ and tdTomato+ cells were manually counted.

### 2.7. Statistical Analysis

A Wilcoxon rank sum test was performed to calculate the significance of results using MSTAT statistical software, version 7.0.1 (available online via https://oncology.wisc.edu/mstat/, accessed on 6 July 2022). This software was written by Dr. Norman Drinkwater, Department of Oncology, McArdle Laboratory for Cancer Research, University of Wisconsin, Madison WI 53705, USA.

## 3. Results

### 3.1. Squamous Cell Carcinomas Contain many Lgr5 Progeny Cells

**Figure 1:**
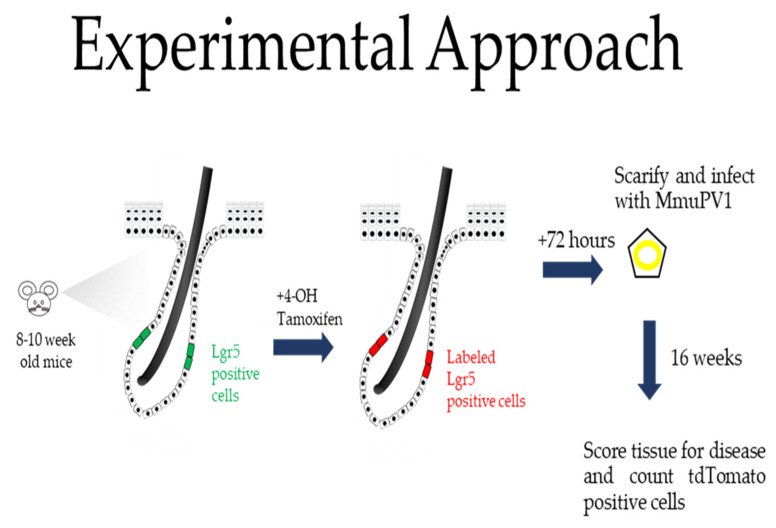
Graphical representation of experimental methods for lineage tracing using Lgr5-eGFP-IRES-CreER^T2^/Rosa26LSLtdTomato (Lgr5 reporter) mice. A representation of a cutaneous hair follicle with approximate location of Lgr5+ progenitor cells is shown. Mice were treated with 4-OH tamoxifen to induce constitutive expression of tdTomato in Lgr5+ progenitor cells and their descendants. Mice were scarified and infected with virus 72 h later, and tissue was harvested 4 months later. The hypothesis being tested is that LGR5+ epithelial progenitor cells preferentially give rise to MmuPV1-induced neoplastic disease. If the hypothesis is correct, then the neoplasia will be tdTomato+.

Experiments were performed on 8–10-week-old Lgr5 reporter mice, as schematically illustrated in Figure 1 and described in detail in the Materials and Methods section. Briefly, their ears were treated topically with 20 µL of 15 mg/mL 4-OH tamoxifen to activate Cre, which is selectively expressed in the LGR5+ epithelial progenitor cells, thereby turning on the expression of tdTomato in the same Lgr5+ epithelial progenitor cells as well as all of their descendants. Then, 3 days later, we infected the ears with MmuPV1 as previously described [28]. This involves lightly scarifying the epidermis to allow for infection by the virus. At 16 weeks post-infection, mouse tissue was harvested for routine histologic analysis and immunofluorescence.

**Figure 2:**
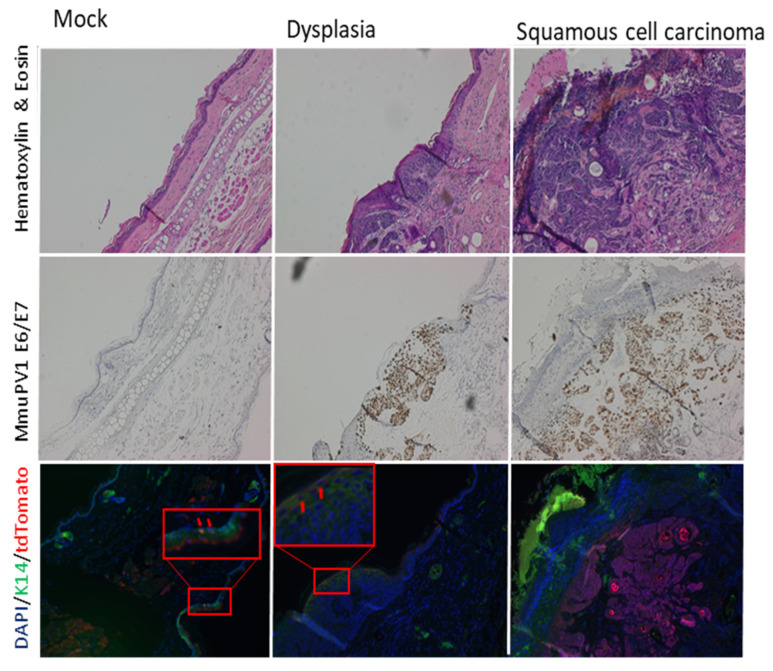
tdTomato+ cells are found abundantly in cSCC. Representative images show that MmuPV1 E6/E7 nucleic acids are found within dysplasia and cSCC. Shown by arrows are infrequent tdTomato+ cells within mock-infected and dysplastic skin (red boxed areas are zoomed digitally for easier viewing). In contrast, large portions of cSCC were composed of tdTomato+ cells. For each sample, serial or near serial slides were used for the different stainings. Images are taken at 200× magnification.

Formalin-fixed, paraffin-embedded sections were stained with hematoxylin and eosin and evaluated by a pathologist (D.B.). MmuPV1-infected sites showed a spectrum of squamous dysplasia/squamous cell carcinoma in situ, which could be flat or exophytic (squamous papilloma), in addition to other infected sites with well-differentiated cutaneous squamous cell carcinoma (cSCC) (Table 1 and Figure 2).

**Figure 3:**
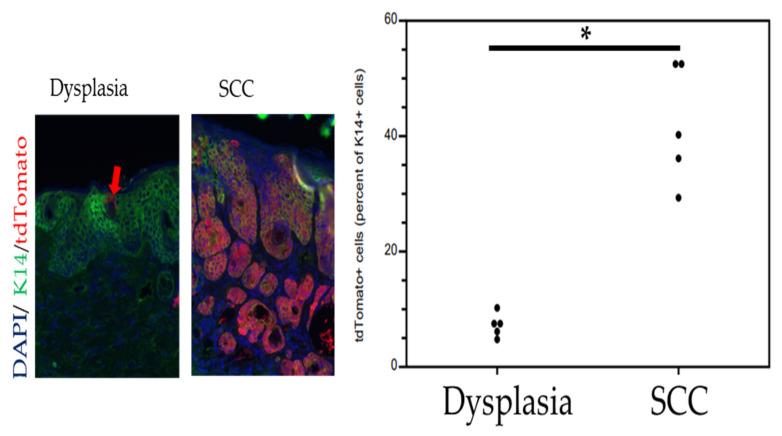
Quantification of tdTomato+ cells at high magnification reveals substantially more tdTomato+ cells in cSCC compared to dysplasia (* *p* = 0.008, two-sided Wilcoxon rank sum test). Images were taken at 400× magnification. Arrow points to examples of rare tdTomato+ cells in dysplasia.

The disease sites contained evidence for the MmuPV1 E6/E7-specific nucleic acids as detected via RNAscope (Figure 2). To look for whether Lgr5+ progenitor cells had been infected and contributed to the lesions, we performed immunofluorescence for tdTomato. There were a few tdTomato+ cells within the inter-follicular epidermis of the mock-infected skin (Figure 2), which is expected as the infection process requires mechanical injury of the epidermis. The migration of Lgr5 progeny outside of the bulge region is well-documented in skin injury [29,30,31]. Based upon the studies cited above, which provided evidence for CRPV infecting the bulge region in rabbits and HPV oncogenes giving rise to lesions consisting of Lgr5 progeny, we predicted that squamous dysplastic lesions/papillomas caused by MmuPV1 would preferentially arise from Lgr5+ epithelial progenitor cells, but this is not what we found. Squamous dysplasia were found to contain only low numbers of tdTomato+ cells (Figure 2). In contrast, cutaneous squamous cell carcinomas (cSCC) appeared to have preferentially arisen from the infection of Lgr5+ epithelial progenitor cells because they had higher percentages of tdTomato+ cells (Figure 2). We quantified the abundance of tdTomato+ cells in five representative lesions scored by the pathologist as dysplasia or cSCC (Figure 3). The percent of tdTomato+ cells was quantified relative to keratin 14 (K14+) cells, a marker abundantly expressed in MmuPV1-infected squamous epithelium. Dysplastic tissue harbored on average 7% tdTomato+ cells while regions histologically representing cSCC harbored on average 42% tdTomato+ cells. This difference was highly significant (*p* = 0.008, two-sided Wilcoxon rank sum test).

### 3.2. Sox9 Is Upregulated in MmuPV1 Infected Sites

**Figure 4:**
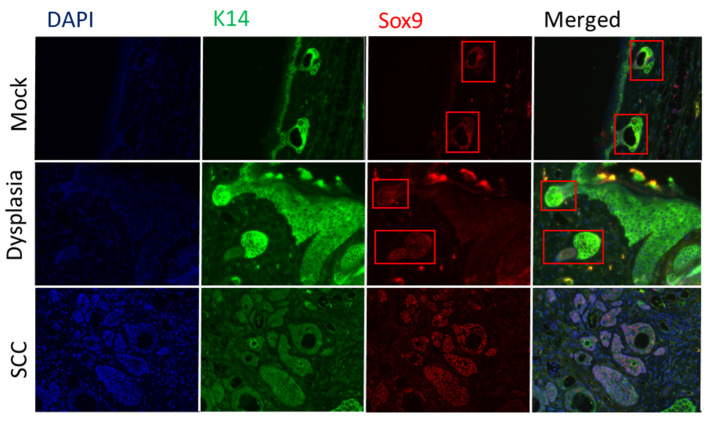
Strong Sox9 staining was restricted to hair follicles in mock-infected skin and dysplasia (see red boxes), with a low level of Sox9 staining found throughout the dysplasia. Higher level Sox9 staining was evident throughout cSCC. Tyramide signal amplification (TSA) was used to detect Sox9. K14 staining intensity was variable amongst SCC. Images were taken at 400× magnification.

Sox9, is a marker for hair follicle bulge resident epithelial progenitor cells and is normally found expressed in the bulge region and surrounding areas [32,33]. Consistent with this, Sox9 staining was limited to the hair follicles within the mock-infected tissue (Figure 4, see boxed areas). In dysplasia, strong Sox9 staining was again seen in the hair follicles associated with dysplasia (Figure 4, see boxed areas); however, light Sox9 staining was also seen throughout the dysplasia as well (Figure 4). Stronger staining for Sox9 was evident throughout the SCCs (Figure 4) and was consistent amongst multiple SCCs stained. These findings are comparable to a previous report indicating that K15+ cells are upregulated in HPV E6/E7-expressing mouse tissue [16]. K15- and Sox9-positive cells were highly overlapping.

### 3.3. Phospho-S6 Is Upregulated in MmuPV1 Infected Sites

**Figure 5:**
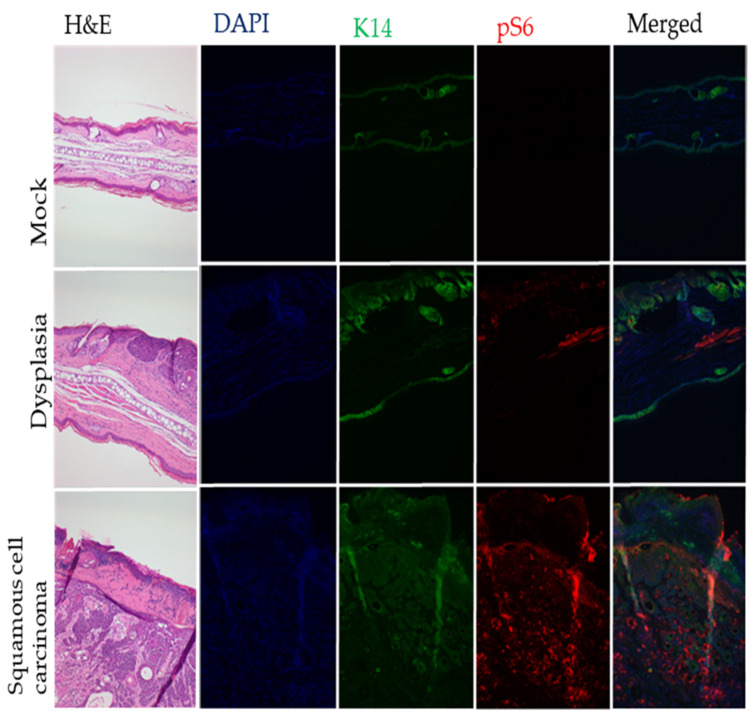
Representative images depict biomarker expression within experimental mice. TSA was used to detect pS6. Note the increased expression of pS6 in cSCC lesions. Images were taken at 200× magnification.

Phospho-S6 (pS6), a marker for the mTOR signaling pathway, is upregulated in HPV-associated cancers and has been extensively used as a biomarker for characterizing progressive neoplastic disease in multiple HPV transgenic mouse models [34,35]. It also was previously reported to be upregulated in oral tissue infected by MmuPV1 [36]. Therefore, we monitored pS6 levels in the different types of tissues from our MmuPV1 infection experiment. In MmuPV1-infected ears, we found that pS6 was substantially upregulated in cSCCs compared to either dysplasia or mock-infected skin (Figure 5).

## 4. Discussion

There is growing evidence indicating that papillomavirus-associated disease arises from an epithelial progenitor cell. With lineage tracing only being possible in mice, this evidence has been restricted to studying disease in HPV-transgenic mice to date [13]. With the discovery of MmuPV1, we were able to observe, in the context of a natural infection model for papillomavirus-induced pathogenesis, that Lgr5+ progenitor cells, which reside in the bulge of the hair follicle, contribute substantially to PV-associated SCCs in mouse skin. We used genetically engineered LGR5 reporter mice to test the hypothesis that LGR5+ epithelial progenitor cells preferentially give rise to MmuPV1-induced neoplastic disease (schematically illustrated in Figure 1). Our results indicate that this hypothesis is correct for squamous cell carcinomas (SCC) arising from MmuPV1 infection but not correct for the precancerous dysplasias. The dysplasias were composed of a minority of tdTomato+ cells indicating that MmuPV1 infects not just LGR5+ epithelial progenitor cells but also other epithelial cells that contribute to long lasting dysplastic lesions (at least 4 months based upon our experiments). Yet, the SCCs arising from these dysplastic lesions were preferentially tdTomato+. We interpret these finding to indicate that the LGR5+ epithelial progenitor cells possess intrinsic properties that support neoplastic progression by MmuPV1, and by inference, non-LGR5+ epithelial progenitor cells are missing intrinsic properties that support neoplastic progression.

Our results are consistent with those arising from the study of HPV transgenic mice that HPV oncogene-induced neoplastic lesions were composed of Lgr5+ progeny cells [13]. In another HPV transgenic model study that there was an expansion of K15-positive cells, which are frequently the progeny of Lgr5+ epithelial progenitor cells [14,15,16,37]. We utilized Sox9, a marker with a highly similar expression pattern to that of K15, to provide further evidence in support of an expansion of the Lgr5+ progeny population and upregulation of stem-like cells within the cSCCs that we observed. This expansion of the Sox9 population of cells indicates a disruption of the balance of stem cell renewal and differentiation. Sox9 has been observed to be both up and downregulated in various cervical cancer cell lines and may either promote or suppress tumor growth [19,38,39]. Sox9 may also bind to the LCR of HPV16 variants [40] and Sox9 and K15 transcripts were found to be downregulated in HPV16 E6 transgenic mouse skin [41]. Thus, it remains unclear what effect HPV oncogenes have on Sox9 expression.

The use of a stem cell or stem-cell-like population to harbor a virus may be advantageous to the virus. These cells are less subject to immune detection and capable of more self-renewal than non-stem cells [37,42]. Furthermore, E7 from cutaneous HPVs 5 and 8 can cause the upregulation of EpCAM and CD44, surface proteins associated with increased stemness [43]. MmuPV1 E6 is capable of binding to MAML1, which inhibits NOTCH signaling, thus inhibiting differentiation [44]. A similar function and mechanism is found in cutaneous HPV types as well [45,46].

Lgr5+ cells have been found within mucosal tissues [47,48,49]. When Lgr5 is overexpressed in cervical cancer cells, there are increases in the tumorigenicity of the cells when grafted onto mice [50]. The knockdown of Lgr5 results in the reduced expression of stem cell-associated factors, notably KLF4 and Oct4 [50]. Lgr5 expression also increases with disease severity in human oral squamous cell carcinoma, although the data were not stratified based on HPV status of the lesions [48,51]. Although Lgr5 has been detected in both the oral mucosa and mucosal epithelium lining the female reproductive tract [47,49], the characterization of stem cells via lineage tracing within these sites is currently not well-known. Thus, Lgr5’s status as a multipotent stem cell population within these tissues in not yet known.

It is important to consider that LRIG1+ epithelial progenitor cells may serve as another population of interest. LRIG1 is found to be upregulated in a variety of HPV-associated cancers [18,52,53,54]. For this reason, we posit that other populations of epithelial progenitor cells may exist that can give rise to cancer associated with those sites, specifically LRIG1+ epithelial progenitor cells. LRIG1+ populations of cells have been found to be increased in HPV8 transgenic disease models [18]. The upregulation of LRIG1+ cells is correlated to HPV+ oropharyngeal and cervical cancers as well [52,53]. This study also found no upregulation of the Lgr5+ progenitor cell population, but ultimately, lineage tracing is needed to conclude the origins of disease in any site [18]. Similar to the Lgr5 population of cells, LRIG1’s status as a multipotent stem cell population within cervical and oropharyngeal tissues is not yet known.

Over the course of time, it may be found that our study is an indication of the importance of Lgr5-positive cells in their cellular contribution to SCCs caused by papillomavirus. Although it has not been found to be upregulated to the same extent, LRIG1 is also found in HPV-associated SCCs, expression. However, this alone does not indicate the origin of disease [52,53]. That being said, it is entirely possible that the upregulation of LRIG1+ cells is reflective of the origin of disease within these tissue sites. Further research is needed to establish if LRIG1, Lgr5, or both contribute to the development of SCCs associated with papillomavirus infection.

## Figures and Tables

**Table 1:** Disease incidence in experimental mice.

Treatment	WNL *	Dysplasia	cSCC
4-OH + Mock	12	0	0
4-OH + MmuPV1	14	14	6

* WNL: within normal limits.

## Data Availability

All relevant data for this study are provided in the tables and figures reported herein.
